# Aspergillosis of Maxillary Sinus's Diagnosis, Management, and Association With COVID-19: A Case Report

**DOI:** 10.7759/cureus.30191

**Published:** 2022-10-11

**Authors:** Dolly Rai, Deepankar Shukla, Nitin D Bhola

**Affiliations:** 1 Oral and Maxillofacial Surgery, Sharad Pawar Dental College and Hospital, Datta Meghe Institute of Medical Sciences, Wardha, IND

**Keywords:** antifungal agent, voriconazole, fungal sinusitis, non-invasive aspergillosis, invasive aspergillosis, covid-19

## Abstract

Aspergillosis is a disease that can manifest itself in both invasive and non-invasive forms. Noninvasive aspergillosis usually affects a healthy host, manifesting as a fungal hyphae cluster or an allergy. In a healthy host, localized invasive infection of damaged tissue is prevalent, but in immunocompromised patients, more extensive infection is often evident, which carries a high mortality rate. Invasive aspergillosis of the paranasal sinuses is a rare condition that is frequently misdiagnosed. Histological analysis and fungal culture are used to make a definitive diagnosis. The purpose of this study is to discuss a case of COVID-19-induced aspergillosis involving the maxillary sinus in an immunocompromised patient, with a focus on early diagnosis because fungi have a predisposition to invade nearby blood vessels and embolize to distant organs, making a delay in treatment which is life-threatening.

## Introduction

Aspergillosis is a disease affecting airway or lung invasion, cutaneous infection, or extrapulmonary spread caused by *Aspergillus* species. *Aspergillus* is a saprophytic mold belonging to the Ascomycetes genus. It is present in several locations, such as soil, cereals, and decomposed vegetation. When airborne resistant spores are discharged, they cause opportunistic fungal infection secondary to candidiasis in terms of prevalence [1]. However, spores are sometimes added to the antrum through oroantral contact. Oroantral contact might happen during a root canal treatment or a tooth extraction. The spores can become pathogenic if they are inoculated into anaerobic sinuses [2]. The lung is the most common site of human infection. The most common fungus that affects the paranasal sinuses is Aspergillus. Human infections have been linked to the species *A. fumigatus, A. flavus, A. glaucus, A. terreus, and A. niger*. Although infectious fungal sinusitis can affect healthy people, Aspergillus fumigatus is most often seen in immunocompromised patients [3].

Fungal sinusitis accounts for around 6% to 9% of all rhinosinusitis cases. The maxillary sinus is the most prevalent place in the head and neck region (87.8%), followed by the sphenoid sinus [4]. Polyps and thick secretions, patients with neutropenia, excessive antibiotic usage, and immunosuppressive medications. Along with radiation therapy, uncontrolled diabetes mellitus, human immunodeficiency virus (HIV) infection, trauma, burns, and corticosteroid treatment. All of these are predisposing factors that encourage fungal infections throughout the sinuses [5].

Aspergillosis can take three forms: 1) Saprophytic, in which the fungus grows without invading viable tissue; 2) Allergic, in which the fungus causes a hypersensitivity reaction to its hyphae or conidia; and 3) Invasive, in which the fungus invades viable tissue and causes extreme necrosis [4]. Aspergillosis of the paranasal sinuses, also known as invasive or fulminant aspergillosis, is a rapidly progressing infection. It tends to affect immunocompromised patients, like patients with diabetes, cancer, or those on steroid or immunosuppressive treatment [6].

Since March 2020, the pandemic has had the biggest global health and social impact [7]. As the fungus has a propensity to infect nearby blood vessels and embolize distant organs, delaying treatment jeopardizes the patient's life and causes life-threatening complications. Our main aim should be early diagnosis and prompt treatment planning. A case of COVID-associated invasive fungal sinusitis, CAIFS (Aspergillosis), is discussed here, with the diagnostic and therapeutic difficulties highlighted. As COVID-19-associated acute invasive fungal rhinosinusitis has been rarely reported yet. We emphasize the necessity of early identification and selection of effective antifungal medication.

## Case presentation

A 72-year-old male reported to the department of oral and maxillofacial surgery, Sharad Pawar Dental College and Hospital, with a history of pain in the right maxillary region approx. since eight days. The patient was diagnosed with diabetes mellitus and hypertension 10 years back. He is on Tab. Gluconorm G2 Forte, Tab. Telmikind AM 40/5 and Tab. Ecosprin AV 75/10. The patient was diagnosed with COVID-19 & was hospitalized for five days. During his stay, insulin therapy, intravenous broad-spectrum antibiotics, dexamethasone, heparin, and other supportive measures were given. After which, he was home quarantined and then tested negative for COVID-19. The patient started experiencing pain in his upper right back region of the jaw one-month post-COVID.

**Figure 1:**
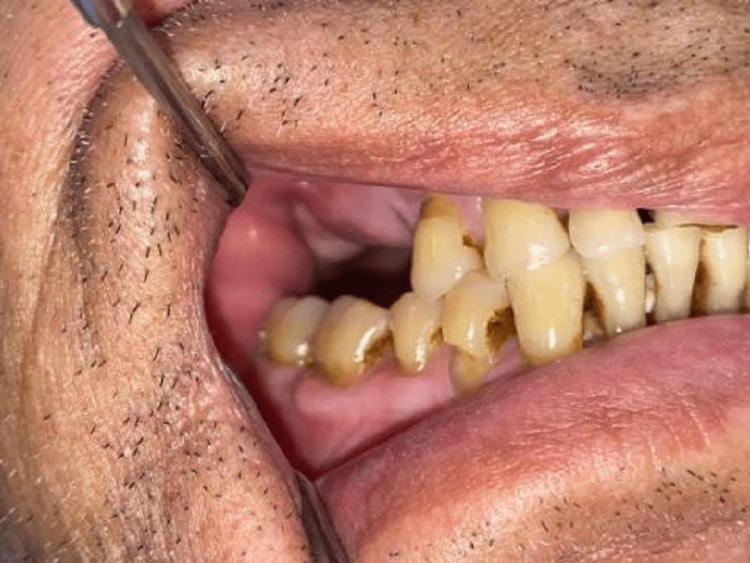
Shows sinus tract was present over the buccal vestibule with 14 & 15. Flabby gingiva and sinus opening with draining pus seen.

On local examination, the pain was gradual in onset, dull aching, and continuous in nature and associated with pus discharge in 14 & 15 regions. The sinus tract was present over the buccal vestibule and palate with respect to 14 & 15 regions. Flabby gingiva and sinus opening with draining pus was seen. The patient underwent extraction with 15, and curettage of the extraction socket was done. He reported to a private clinic where he was advised root canal treatment (RCT) of 14 (Figure 1).

**Figure 2:**
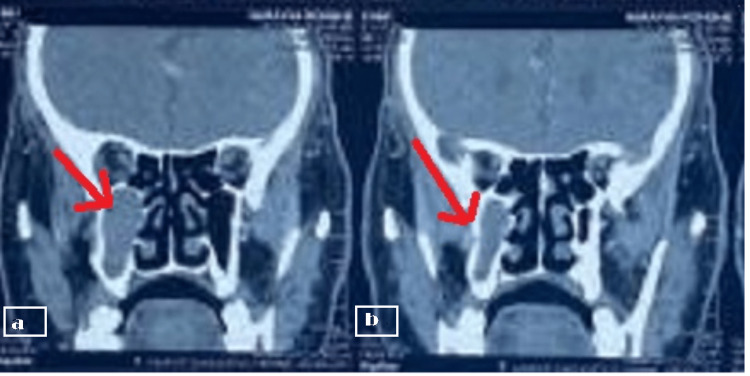
a) The arrow shows mild enhancing mucosal thickening in the right maxillary sinus b) The arrow shows associated rarefaction of the floor of the right maxillary sinus.

Diagnostic assessment following root canal treatment of the respective teeth, Computed Tomography (CT) of paranasal sinuses was done. Reports suggest mild enhancing mucosal thickening in the right maxillary sinus, along with associated rarefaction of the right maxillary sinus floor with lucencies in the alveolar process of the maxilla (Figure 2). The granulation tissue obtained after the extraction of 15 was sent for microbiological examination on the Potassium hydroxide (KOH) mount. Reports revealed growth of *Aspergillus flavus.* He was then referred to the Department of the ear, nose, and throat (ENT) for direct nasal endoscopy examination for further treatment planning. Nasal endoscopy was suggestive of right osteomeatal complex blockage with polypoidal growth.

In therapeutic interventions, the patient was kept on strict follow-up because of any disease progression for at least five years. Subsequently, he was placed in conservative management. Tab. Voriconazole 200mg thrice daily for 14 days & Tab. Dalacin C 300mg twice daily. But the patient was lost to follow-up.

## Discussion

Aspergillomas, named after the Aspergillus species, are the most commonly encountered fungus. Aspergillus can enter the oral mucosa through the soft tissue lining of the maxillary sinus in aspergillosis [8]. It can spread to the bone, cause a palatal infarction, appear in the oral mucosa, and migrate to other organs in the body. In immunocompromised or diabetic individuals with severe sinusitis, nasal septal mucosa irritation, unexplained fever, or cough, invasive fungal sinusitis can be considered. 

Intracranial and intra-orbital extensions cause an increase in surgical morbidity. Cavernous sinus syndrome and cranial nerve deficits may result from extensions across the sphenoidal sinuses. Carotid artery collapse, mycotic aneurysms, and cavernous sinus thrombosis are also possible risks for patients [9].

Washburn et al. reported individuals with invasive illness who were subjected to thorough testing; these examinations included measurements of absolute neutrophil and monocyte counts, the phagocytic and fungicidal activity of peripheral blood monocytes, total lymphocyte counts, natural killer cell activity, and immunoglobulin levels." In the investigation, no consistent deficits were discovered. The most prominent factors that predispose are neutropenia and glucocorticoid use. The disease is characterized by vascular penetration, which leads to infarction and tissue necrosis. A biopsy is needed to confirm the diagnosis. Choi et al. recorded a case of paranasal sinus aspergillosis that presented as optic neuritis. Antibiotics should be used with caution, and judicious testing should be considered in such patients [10].

In contrast to invasive fungal sinusitis, in the middle-aged female population, fungus balls have been observed to be common. The pathophysiology is not completely understood. Inhaled spores, like a saprophytic fungal infestation, are a commonly established method of fungal entry to the sinus. Earlier surgery or mucosal injury could be the cause. Furthermore, there is a link between maxillary fungal balls and recent dental treatment, Iatrogenic oroantral communication, and dental fillings, in particular. This is because certain components of treatment sealers (such as Zinc Oxide) may encourage fungal development. However, following the initial intervention, this can take years to manifest. The link has been demonstrated to be extremely strong. Patients who had previous dental work had rates as high as 89.2 percent, according to several studies [11]. In the treatment of patients who are suspected of having dental disease, panoramic dental imaging should be a requirement. The production of fungal balls is hypothesized to be caused by anatomical variance and blockage at the osteomeatal complex. Other types of fungal sinus illness may coexist with fungus in the sinuses.

For detecting calcified aspergillus colonies, CT and MRI scans are valuable diagnostic aids. MRI has benefits in terms of demonstrating soft-tissue and vascular invasion, as well as the ability to distinguish between sinus inflammatory tissue, mycetoma, and neoplasm. An ophthalmologist can confirm central retinal artery occlusion, and prompt treatment was required within the first 90 minutes. On this basis, an ophthalmologist who specializes in related diseases should be consulted. Mucormycosis, pseudomonas orofacial lesions, and Wegener's granulomatosis must all be included in the differential diagnosis of fulminant aspergillosis.

Surgical debridement and antifungal injection are the principal treatments for aspergillosis. If complete removal is not possible, antifungal agents, especially voriconazole, should be used as a first-line treatment. Voriconazole is the preferred therapeutic agent for the treatment of invasive aspergillosis, with liposomal amphotericin B and isavuconazole as alternatives. While systemic amphotericin B deoxycholate therapy was once thought to be the best option, studies have shown that voriconazole, a triazole antifungal agent, is more effective [12]. In the case of chronic renal disease, liposomal amphotericin B can be begun right away as a second-line treatment [13].

During the pandemic, severe acute respiratory syndrome coronavirus 2 (SARS-CoV-2) caused the most serious health consequences. Since March 2020, there has been a significant increase in global, economic, and social consequences. According to Salehi et al. (2020), patients with or without a history of diabetes mellitus, hypertension, hospitalization to intensive care units, or use of immunosuppressant drugs or corticosteroids are considered high-risk COVID-19 patients [14]. These patients are most likely to develop a fungal infection in the post-COVID period [14]. Voriconazole plus caspofungin or amphotericin B plus itraconazole for skull base aspergillosis is recently being thought to represent a breakthrough in the treatment of invasive aspergillosis.

## Conclusions

Invasive maxillary sinus aspergillosis should be considered when maxillary sinusitis does not respond to normal conservative antibiotic therapy in immunocompromised patients. Since fungal infections are uncommon, they can be difficult to diagnose and treat for those who are unfamiliar with their clinical manifestations. This case report offers some therapeutic and diagnostic hints for treating patients safely and reliably while avoiding any inability. As a result, we stressed the importance of early detection to prevent the high costs of care; timely diagnosis and treatment are necessary for decreasing the high morbidity and mortality rates associated with this debilitating condition.
